# Anaemia, iron homeostasis and pulmonary hypertension: a review

**DOI:** 10.1007/s11739-020-02288-1

**Published:** 2020-02-10

**Authors:** Thomas Sonnweber, Alex Pizzini, Ivan Tancevski, Judith Löffler-Ragg, Günter Weiss

**Affiliations:** 1 Department of Internal Medicine II, Medical University Innsbruck, Innsbruck, Austria; 2 Christian Doppler Laboratory for Iron Metabolism and Anaemia Research, Medical University Innsbruck, Innsbruck, Austria

**Keywords:** Anaemia, Pulmonary hypertension, Iron deficiency, Cardiovascular disease, Hypoxia

## Abstract

Anaemia is a highly prevalent condition, which negatively impacts on patients’ cardiovascular performance and quality of life. Anaemia is mainly caused by disturbances of iron homeostasis. While absolute iron deficiency mostly as a consequence of chronic blood loss or insufficient dietary iron absorption results in the emergence of iron deficiency anaemia, inflammation-driven iron retention in innate immune cells and blockade of iron absorption leads to the development of anaemia of chronic disease. Both, iron deficiency and anaemia have been linked to the clinical course of pulmonary hypertension. Various mechanistic links between iron homeostasis, anaemia, and pulmonary hypertension have been described and current treatment guidelines suggest regular iron status assessment and the implementation of iron supplementation strategies in these patients. The pathophysiology, diagnostic assessment as well as current and future treatment options concerning iron deficiency with or without anaemia in individuals suffering from pulmonary hypertension are discussed within this review.

## Introduction

Anaemia is one of the most important health problems worldwide. It is associated with physical and mental developmental disorders, impaired cardiovascular performance along with reduced quality of life and often linked to increased morbidity and mortality specifically as a co-morbidity across a vast range of disease entities [1–3]. Anaemia affects up to 25% of the world’s population with vast locoregional differences. Pre-school children, pregnant and premenopausal women, elderly and individuals contracted with chronic diseases are at a higher risk [1, 2]. The majority of all cases of anaemia are caused by imbalances of iron homeostasis, given that iron is essential for the formation of haemoglobin. The underlying problem is the insufficient availability of iron for erythropoiesis, which can be caused by two different mechanisms, absolute and/or functional iron deficiency (ID). Absolute ID causes iron deficiency anaemia (IDA), the most frequent form of anaemia, whereas functional ID leads to the development of anaemia of chronic disease (ACD), the second most frequent cause of anaemia in the world [1, 2].

Recently, the association of anaemia, altered iron homeostasis and the pathobiology of various forms of pulmonary hypertension (PH) have attracted attention, because treatment of ID with or without anaemia in PH related diseases including congestive heart failure improved patients` exercise tolerance, quality of life, hospitalization rates and mortality [4–6]. We herein shed light on the complex interaction of iron homeostasis, anaemia and PH, and aim to give guidance on how to select PH patients who might benefit from iron supplementation.

## Disorders of iron homeostasis and anaemia

**Fig. 1:**
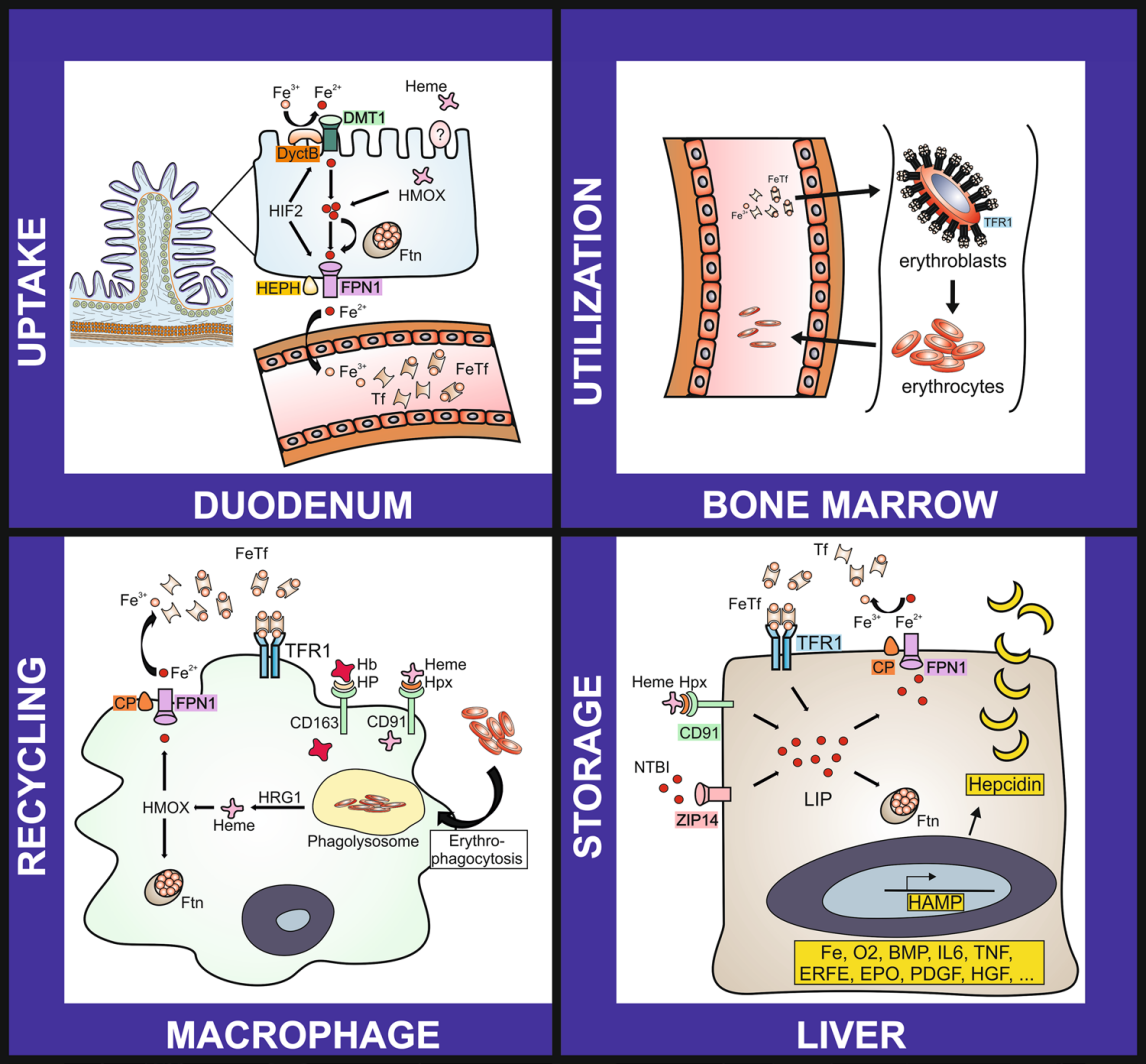
Regulation of iron homeostasis in the human body

Iron (Fe) is an essential element of life, which participates in numerous physiological processes, including immune-surveillance, oxygen transfer, cellular proliferation and various cellular metabolic activities such as mitochondrial respiration [7]. Whereas unbound iron is highly toxic due to its oxidoreductase function, which may foster the production of reactive oxygen (ROS) and nitrogen species (RNS), a lack of iron is associated with detrimental effects, such as impaired haemoglobin synthesis and mitochondrial dysfunction [8, 9]. For this reason, human iron homeostasis displays a strictly adjusted transport, recycle and storage system [7] (Fig. 1).

Nutritional heme or non-heme bound iron acquisition takes place in the duodenum where divalent metal transporter 1 (DMT1) or a heme receptor at the luminal and ferroportin 1 (FPN1) at basolateral site act in concert with oxidoreductases (duodenal cytochrome B (DcytB) and hephaestin (HEPH), respectively) to shuttle ionic iron from the enteral lumen to the circulation. Additional, transport systems foster the acquisition of protein bound iron. Hypoxia-inducible factor 2 (HIF2) and ferritin (Ftn) are essential for the regulation of nutritional iron absorption within the gut. In the circulation iron is mainly bound to transferrin (Tf). Tf delivers the metal to target cells, which acquire iron loaded-Tf via receptor mediated endocytosis, involving the transferrin receptor 1 (TfR1). Whereas nutritional iron uptake is relevant for the long term adaption of iron homeostasis, the daily iron need is met via recycling of the metal from senescent red blood cells. The latter is achieved by the mononuclear phagocyte system (MPS). Quantitatively erythropoiesis consumes the major amount of the daily iron turnover, because iron is needed for heme synthesis and permits proliferation of hematopoietic cells. Thus, erythroblasts demonstrate high expression levels of TfR1 and other iron uptake systems. Hepatic iron storage serves as a buffer system and releases or sequesters iron in chases of higher iron demand or iron overload, respectively. Additional abbreviations: BMP, bone morphogenetic protein; CP, ceruloplasmin; EPO, erythropoietin; ERFE, erythroferrone; Fe, iron; FeTf, iron loaded transferrin; HAMP, hepcidin antimicrobial peptide; Hb, haemoglobin; HFE, human hemochromatosis gene; HGF, hepatocyte growth factor; HMOX 1, heme oxygenase 1; HP, haptoglobin; Hpx, hemopexin; HRG1, haem transporter 1; IL6, interleukin 6; LIP, labile iron pool; NTBI, non-transferrin bound iron; O2, oxygen; PDGF, platelet-derived growth factor; TNF, tumor necrosis factor; ZIP14, solute carrier family 39 zinc transporter.

In situations of increased iron demand, e.g. acute blood loss or increased haematopoiesis following hypoxic challenge, comprehensive mechanisms adjust iron handling. A central regulator is hepcidin, a small acute phase peptide encoded by the HAMP gene, which controls cellular ferroportin (FPN) expression [10]. FPN is the only known cellular iron exporter, thus hepcidin mediated lysosomal degradation of FPN vastly affects systemic iron availability. A rise in hepcidin results in sequestration of iron within iron storage cells and it constrains duodenal iron acquisition [10]. Consequently, high hepcidin levels reduce systemic iron availability and results in hypoferremia. This mechanism is considered to be a pivotal part of nutritional immunity as it prevents the assimilation of iron by extracellular bacteria, fungi and protozoa, for whom the metal is essential for pathogenicity and proliferation [11]. On the contrary, chronic inflammation in the absence of infection is also associated with an increase of hepcidin and may result in ID [12, 13].

Various alterations of iron metabolism are associated with different pathological features and consequences. Absolute (true) ID, which arises from iron loss (e.g. bleeding), insufficient nutritional iron uptake or malabsorption (mainly found due to under- and/or malnutrition in less developed countries with high poverty rates), or a higher iron demand (e.g. menstruating or pregnant women; children and adolescents), results in an iron-restricted erythropoiesis and may progress into overt IDA [1]. Contrary, the term functional (relative) ID describes a depletion of iron from certain tissues and cells, whereas total body iron content is not reduced [14]. The latter ensues the emergence of ACD. Functional ID is often related to hepcidin overexpression, and due to the previously described inflammation dependent upregulation of hepcidin, it is most frequently found in diseases associated with chronic inflammatory processes [14, 15]. Whereas absolute ID may be identified rather easily due to markers of depleted iron storage and/or typical features of IDA, such as microcytosis of red blood cells, the diagnosis of functional ID or combined forms of absolute and relative ID may be rather complicated [12]. These complex disorders of iron homeostasis are mainly found due to multifactorial inputs such as chronic inflammation in combination with bleeding episodes, malnutrition and/or malabsorption and may result in complex forms of anaemia (e.g. IDA + ACD or unclassifiable anaemia).

## Pulmonary hypertension

PH is a frequent clinical problem, which is found in approximately one per cent of the global population, and its prevalence increases with age, reflected by the estimation that above the age of 65 years approximately 10% of individuals suffer from PH [16]. The European Society of Cardiology (ESC) and European Respiratory Society (ERS) guidelines define PH by a mean pulmonary arterial pressure (mPAP) of ≥ 25 mmHg at rest measured via right heart catheterization [17]. This currently used hemodynamic definition of PH has been recently re-evaluated at the 6th World Symposium on Pulmonary Hypertension (WSPH), and may be adapted in future guidelines. Data suggest that the mPAP found in healthy subjects at rest is 14 ± 3.3 mmHg, resulting in a definition of PH if a mPAP greater than 20 mmHg is present [18, 19].

The ESC/ERS categorize PH into five different World Health Organization (WHO) groups or entities [17]. WHO group 1 encompasses patients with idiopathic pulmonary arterial hypertension (IPAH), as well as heritable forms (HPAH), drugs or toxin-induced PH and PH associated with connective tissue disease, portal hypertension, congenital heart disease, human immunodeficiency virus infection or schistosomiasis. Additionally, WHO group 1 includes individuals with pulmonary veno-occlusive disease and/or capillary haemangiomatosis as well as persistent PH of the newborn. WHO Group 2 covers PH in patients with left heart disease, and WHO group 3 summarizes individuals with PH due to lung disease and/or chronic hypoxia. WHO group 4 encompasses patients with PH caused by thromboembolic events, also termed chronic thromboembolic pulmonary hypertension (CTEPH), or other pulmonary artery obstruction. Finally, WHO group 5 compromises individuals with a PH of unclear or multifactorial mechanism [17]. Additionally, according to hemodynamic features PH is subdivided into pre-capillary, post-capillary, and combined pre- and post-capillary PH (Table 1).

**Table 1:** Hemodynamic definitions of pulmonary hypertension (adapted from [17])

Definition	mPAP (mmHg)	PAWP (mmHg)	PVR (WU)	DPG (mmHg)
Pre-capillary PH	≥ 25	≤ 15	> 3	≥ 7
Post-capillary PH	≥ 25	> 15	≤ 3	< 7
Combined pre- and post-capillary PH	≥ 25	> 15	> 3	≥ 7

*PH* pulmonary hypertension, *mPAP* mean pulmonary arterial pressure, *PAWP* pulmonary arterial wedge pressure, *PVR* pulmonary vascular resistance, *DPG* diastolic pressure gradient (DPG = diastolic PAP (dPAP) − PAWP), *WU* wood units

Pre-capillary PH is mainly found in PH WHO groups 1, 3, and 4, whereas post-capillary PH is a typical feature of WHO group 2 [20]. Combined forms of pre- and post-capillary PH are present in patients with complex diseases, as seen in WHO group 5, or multi-morbid individuals, e.g. patients with chronic obstructive pulmonary disease (COPD) and heart failure. Quantitatively most cases of PH demonstrate features of PH due to left heart disease (WHO group 2) and/or PH due to lung diseases (WHO group 3), whereas a substantially smaller group of patients compromises PAH or CTEPH.

The pathobiology of pre- and post-capillary PH involves vastly different mechanisms. Pre-capillary PH of WHO group 1 is mainly caused by a chronic progressive remodelling of pulmonary arteries and capillaries, resulting in vessel lumen narrowing and obstruction as well as the formation of complex vessel lesions, also termed plexiform lesions, thrombotic lesions and a partial loss of pre-capillary arteries [20]. This impairment of the pulmonary vascular bed is accompanied by an increase of pulmonary vascular resistance and pulmonary blood pressure as well as a rise of right heart pressure. An acute elevation of right heart pressure, as seen in acute pulmonary embolism, may result in acute right heart failure, whereas chronic pressure elevation induces a dilation and remodelling of the right heart leading to chronically progressive right heart failure. WHO group 4 PH has a distinguished pathobiology as thromboembolic events are the main driver of pulmonary vessel obstruction in this group. Interestingly, even though mechanical obstruction is the main driver of pre-capillary disease in these individuals, vascular remodelling occurs as well, if the mechanical obstruction persists for a longer time [21]. Pre-capillary PH is also frequently found in patients suffering from chronic hypoxia due to pulmonary diseases, such as in individuals with severe COPD [22]. In these patients, mPAP and PVR are typically mild to moderately elevated, whereas patients with IPAH or CTEPH may present with distinctly higher mPAP and pulmonary vascular resistance (PVR).

Post-capillary PH and mixed pre-post-capillary PH feature the involvement of post-capillary pulmonary vessels caused by parenchymal destruction, inflammatory processes and chronic elevation of post-capillary pressure as a consequence of impaired left heart function [23]. Additionally, hemolysis and decompartmentalisation of haemoglobin play a pivotal role in the emergence of PH in WHO group 5 patients suffering from haemoglobinopathies associated with hemolytic anaemia [24]. In this context it is noteworthy that these diseases are also associated with vast alterations of iron homeostasis. For instance, beta-thalassemia, a hereditary disease associated with hemoglobin deficiency and anaemia, causes massive iron overload which contributes to the development of pulmonary hypertension [25, 26].

The terms pre- and post-capillary PH as well as the term PAH have to be precisely utilized in the clinical practice. PAH specifically defines WHO group 1 pre-capillary pulmonary hypertension and, as depicted previously, post- and combined pre-post-capillary PH are related to different pathomechanisms, thus urge differential treatment approaches. Consequently, PAH specific medications are primarily indicated in PAH and CTEPH, whereas other forms of PH may not benefit from such treatment or even deteriorate after implementation of specific PAH drugs.

Despite these established general concepts of PH pathobiology and the expansion of knowledge in the field within recent years, many questions concerning the pathobiology of PH await clarification. An extensive description of the current knowledge of PH pathology exceeds the scope of this review and the interested reader may be referred to excellent recent reviews on this topic (e.g. [20]). Herein, we focus on the fascinating intersection of iron homeostasis and anaemia with the pathobiology of PH.

## Interconnection of pulmonary hypertension, iron homeostasis and anaemia

Evaluations of PH cohorts demonstrate a high frequency of ID and/or anaemia in PH patients, as approximately 40–60% of PH patients demonstrate ID and one-third of all PH patients suffer from anaemia [27, 28]. Interestingly, both ID and anaemia significantly affect the morbidity and mortality of PH patients [27, 29]. Current evidence suggests a significant role of iron handling in the pathogenesis and clinical outcome of both, pre- and post-capillary pulmonary hypertension [4, 28, 30–32]. First, chronic progressive remodelling of pulmonary vessels, resulting in a loss of pre-capillary pulmonary arteries and a pattern of pulmonary vessels rarefication accompanied by progressive right heart disease is a key feature of PAH and other forms of PH [20]. Vascular remodelling results from complex pathobiology and involves an accumulation of pulmonary artery smooth muscle cells (PASMCs), pulmonary arterial endothelial cells (PAECs), fibroblasts, myofibroblasts and pericytes. Interestingly, the FPN/HAMP axis and the haemoglobin scavenger transporter CD163 are important regulators of cellular function and proliferation of PAECs and PASMCs [33, 34]. Accordingly, iron dyshomeostasis contributes to pulmonary vascular endothelial dysfunction. Endothelial dysfunction is characterized by an impairment of pulmonary vessel dilation, particularly mediated by overexpression of endothelin 1 (ET1) and suppression of the nitric oxide (NO)/NO-synthase (NOS) pathway. Of note, iron affects that pathway by reducing the transcriptional expression of inducible NOS, and iron loading of patients has been linked to an impaired end-diastolic dilation along with reduced circulating nitrate levels [35, 36]. Endothelial dysfunction is further linked to NO break down via increased activity of arginase in PAECs [37]. Additionally, perivascular recruitment of B- and T-lymphocytes, macrophages, mast cells, dendritic cells and imbalances of T-regulatory cell function, as well as a T-helper 17 cell immune polarization, are implicated in PH pathophysiology [38, 39]. Perivascular inflammation is characterized by a local overexpression of a plethora of chemokines (e.g. macrophage migration inhibitory factor (MIF), cytokines [e.g. interleukin (IL) 1, IL6, IL10 and tumour necrosis factor-alpha (TNFalpha)] and growth factors [e.g. transforming growth factor-beta (TGFbeta) and platelet-derived growth factors (PDGFs)] [20, 38, 39]. Consequently, vasoconstriction, pro-thrombotic activities as well as an impairment of angiogenesis and tissue repair contribute to the distinct pulmonary vascular phenotype in PH patients [38, 39]. Interestingly, almost all of these mentioned mechanisms, involved in PH associated vascular remodeling and endothelial dysfunction, are linked to iron homeostasis, as iron exerts pivotal immunomodulatory effects, including the control of cytokine production and ROS/RNS generation by immune effector cells, the regulation of T cell polarization, and the functionality of the NO/NOS pathway as well as ET1 expression [8, 11, 34, 40, 41].

Second, heritable forms of PAH are associated with variants in the TGFbeta receptor family and have revealed an important role of bone morphogenetic protein receptor type 2 (BMPR2) mediated signalling in the pathophysiology of PH [42–44]. For instance, treatment with TGFBRII-Fc, a selective TGF-beta1/3 ligand trap, mitigates pulmonary vascular remodelling and PH in monocrotaline-treated rats, SU5416/hypoxia-treated rats, and SU5416/hypoxia-treated mice [45]. Additionally, enhancement of BMPR2 function in PAECs reverses the PAH phenotype of mice with heterozygous R899X BMPR2 mutations [44]. Bone morphogenetic proteins (BMPs) interact with various receptors of the TGFbeta receptor family and induce small mothers against decapentaplegic (SMAD) signalling, which facilitates various biological effects including proliferative and immune-modulatory functions [46]. In PAH BMPR2 function also intersects with inflammatory pathways as a reduced BMPR2 activation results in perivascular recruitment of macrophages via the induction of growth factor and granulocyte–macrophage colony-stimulating factor (GM-CSF) [47]. Further, the binding of the iron dependent pro-inflammatory cytokine MIF to its receptor CD74 was shown to induce pro-inflammatory factors and cell adhesion molecules, such as ICAM1, VCAM1 and E-selectin in PAECs derived from IPAH patients [38]. Thus, MIF/CD74 interaction contributes to vascular inflammation and endothelial dysfunction, two primary instigators of PAH [38]. Noteworthy, BMP/SMAD signalling is central in the systemic regulation of iron homeostasis, as several BMPs are strong inducers of hepcidin [7, 46].

Another link between BMPR2 signalling, perivascular inflammation and iron homeostasis is established for the IL6/signal transducer and activator of transcription 3 (STAT3) pathway [48]. The expression of IL6, a multifunctional pro-inflammatory cytokine and anti-inflammatory myokine is induced by iron depletion [49]. Vice versa, IL6 stimulates hepcidin expression via the STAT3 pathway thereby causing intracellular iron sequestration [50]. Notably, PH related BMPR2 deficiency increases intracellular phospho-STAT3 levels in murine and human PASMCs. This process may be escalated via additional stimuli, as chronic lipopolysaccharide (LPS) application triggers PH, and BMPR2 heterozygote mice have a more prominent IL6 and keratinocyte chemoattractant (KC) response to LPS challenge as compared to wildtype littermates [48]. To conclude, a lack of BMPR2 function or expression may promote alterations of iron homeostasis in PH patients, and iron dyshomeostasis per se alters BMPR2 related singalling, which contribute to the pivotal pathophysiological features of endothelial dysfunction and perivascular inflammation in PH patients.

Interestingly, the HAMP/FPN axis is also implicated in the emergence of a vasoconstrictive milieu in PAH. Both PASMC and PAECs express FPN, and IL6 and/or hepcidin treatment resulted in FPN suppression and proliferation of PASMCs [33]. In addition, mice with a smooth muscle-specific knock-in of the hepcidin-resistant FPN isoform fpnC326Y develop PAH and right heart failure [34]. This process could be linked to an overexpression of FPN, intracellular ID and increased expression of the vasoconstrictor ET1 by PASMCs. Overexpression of ET1 is a key feature in the pathogenesis of PAH. Accordingly, ET1 levels are increased in patients suffering from PAH, and high serum levels of ET1 are associated with an unfavourable clinical outcome [51]. Interestingly, the PAH phenotype of fpnC326Yfl/fl SMMHC-CreERT2 + mice was reversible upon treatment with intravenous iron. Furthermore, intracellular ID in PASMCs contributed to vascular remodelling [34]. Thus, a local dysregulation of iron homeostasis in pulmonary vessels likewise contributes to the emergence of PAH, which, however, is not necessarily associated with systemic alterations of iron homeostasis. Interestingly, a similar mechanism of a locally regulated HAMP/FPN axis was found in cardiomyocytes, which may explain why dysregulation of iron homeostasis is also associated with poor clinical outcome in post-capillary PH patients with chronic heart disease [52].

Third, acute or chronic hypoxic challenge ensues pulmonary hypertension and results in pulmonary vascular remodelling [53]. The adaption to hypoxic challenge is mainly facilitated by hypoxia-inducible factors (HIFs), which are nuclear transcription factors existing in three different isoforms, namely HIF-1alpha, HIF-2alpha and HIF-3alpha [54]. Hypoxia ensues a HIF mediated increase of erythropoietin (EPO) formation by the kidneys, which induces erythropoiesis and hampers the systemic expression of hepcidin [55]. Hypoxia also induces platelet-derived growth-factor BB (PDGF-BB), erythroferrone (ERFE), matriptase-2 and growth differentiation factor 15 (GDF-15), which mediate hepcidin suppression [7, 56–58]. Consequently, hypoxia increases duodenal iron absorption facilitating a sufficient iron supply for hypoxia triggered stress erythropoiesis [59]. Additionally, HIFs bind to multiple other target genes that are implicated in the expression of growth factors such as vascular endothelial growth factor (VEGF) and TGFbeta which then induce ET1 expression and adapt vessel tension. The expression of HIFs is linked to both, the availability of iron and oxygen. Iron modulates HIF expression via interaction of IRPs with IREs within HIF mRNA [7]. Further, intracellular ID hampers the degradation of HIFs by limiting the activity of iron-dependent prolyl hydroxylases (PHDs) and their interaction with the von Hippel-Lindau (VHL) factor [59, 60]. Notably, these regulatory mechanisms of iron are essential for the adaption to hypoxic challenge and the emergence of hypoxia triggered PH [53]. Accordingly, ID, application of the iron chelator desferrioxamine (DFO) or iatrogenic blood drain exaggerated hypoxia driven PH. [53, 61]. These observations in humans are underscored by mechanistic data from rodents showing that IRP1 deletion causes PH in mice through derepression of HIF2alpha [60, 62].

Notably, hypoxia-mediated PH is also linked to pro-inflammatory processes, and elevated serum levels of pro-inflammatory cytokines, such as IL1, IL6, MIF, leptin and TNFalpha, are associated with hypoxia, ID and poor prognosis in PH patients [38, 63–66]. In this context, mice with hypoxia-induced PH display an increased interleukin 1 beta (IL1beta) expression [65]. The interaction of IL1beta with the IL1 receptor (IL1R) mitigates nuclear factor-kappaB (NF-kappaB) activation [65]. NF-kappaB is an iron-dependent transcription factor, which per se induces IL1, IL6 and TNFalpha synthesis. Elevated serum IL6 levels, alterations of STAT3 signalling, ectopic upregulation of the IL6R in PASMCs and IL6 promoted perivascular infiltration of macrophages were all associated with the development of PH [48, 67]. Accordingly, hypoxia-induced TNFalpha expression is also linked to PH. TNF inhibits BMPR2 expression and promotes cleavage of BMPR2 via the disintegrin and metalloproteases ADAM10 and ADAM17 in PASMCs [66]. Consequently, BMP signalling is subverted and BMPs, such as BMP6 and BMP9, primarily activate the ALK2/ACTR-IIA signalling axis, which favours PASMC proliferation and vascular remodelling in heritable PAH [44, 66]. Additionally, TNFalpha increases NOTCH2 signalling, which blocks BMPR2 expression [66]. Both IL6 and TNFalpha, also induce ROS/RNS production [55]. A contributing role of ROS to the PH pathobiology is suggestive as induction of ROS by dasatinib induces endothelial cell dysfunction and increased susceptibility to pleural effusion in a PH rodent model [68]. Interestingly, production of ROS/RNS is closely linked to iron availability, as enzymatic processes leading to the generation of ROS/RNS, such as the Fenton–Haber–Weiss reaction, are iron-dependent [69]. Additionally, ROS generation and ID are associated with an impairment of mitochondrial function and have been linked to cardiovascular diseases [70]. For instance, reduced mitochondrial superoxide dismutase 2 (SOD2) levels were found in PASMCs of PAH patients and blunted SOD2 function ensues an apoptosis-resistant phenotype of PASMCs, which may foster PASMC accumulation in pulmonary vessels of PH patients [71]. In line with this, the mitochondrial iron-sulfur cluster scaffold Protein NFU1 is linked to heritable forms of PAH, and the autosomal recessive inheritance of the NFU1 mutation G208C causes PAH in approximately 70% of affected cases [72].

**Fig. 2:**
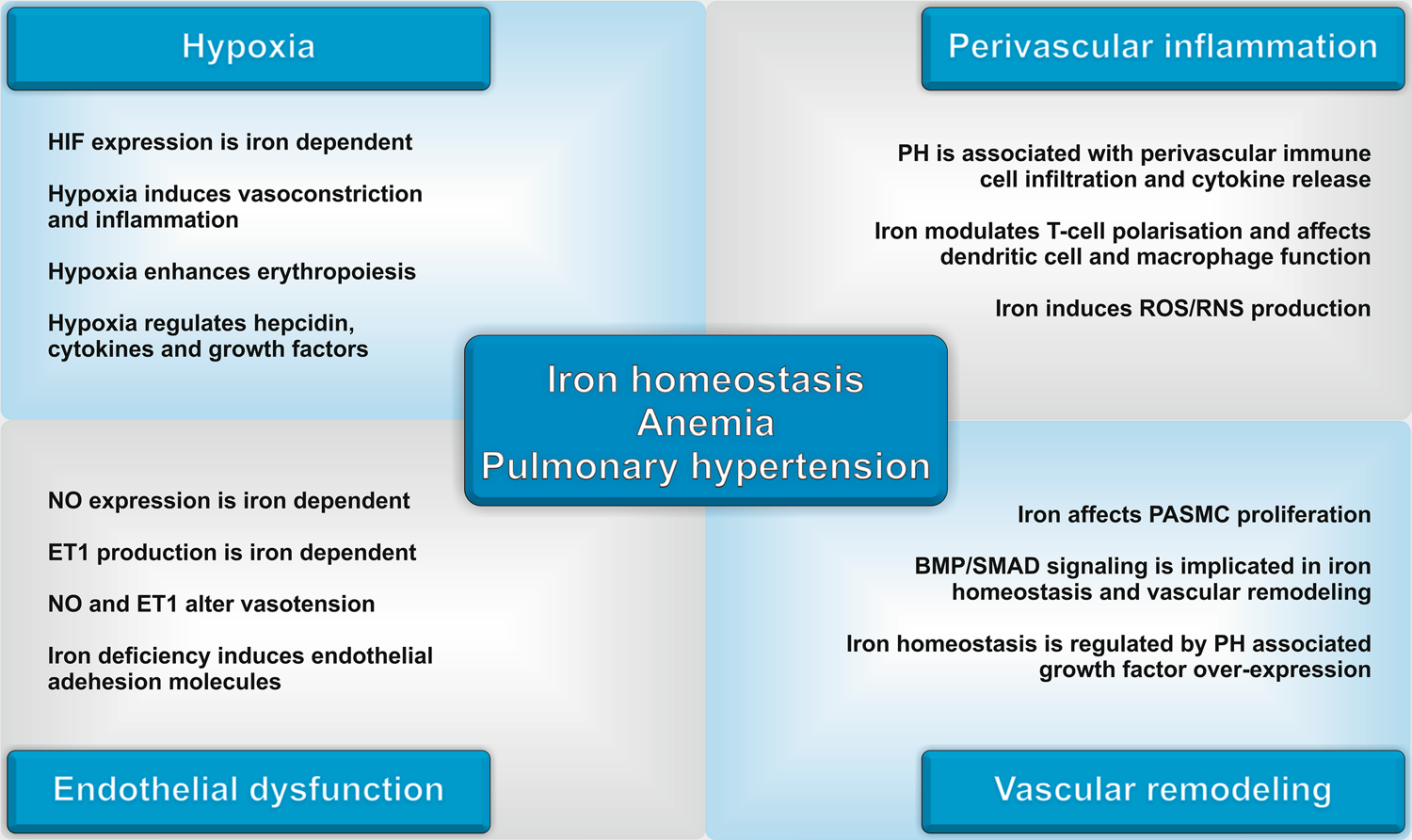
Current concepts of the interconnection of iron homeostasis, anaemia and pulmonary hypertension. *HIF* hypoxia-inducible factor, *NO* nitric oxide, *ET1* endothelin 1, *PH* pulmonary hypertension, *ROS* reactive oxygen species, *RNS* reactive nitrogen species, *BMP* bone morphogenetic protein, *SMAD* small mothers against decapentaplegic

To conclude, hypoxic stress, alterations of mitochondrial function, ROS/RNS production, blunted NO/NOS signalling, inflammatory processes, BMP/SMAD/HAMP as well as IL6/STAT3/HAMP signalling are all implicated in both, iron handling and the emergence of PH (Fig. 2). Despite these numerous mechanistic links between iron homeostasis and pulmonary hypertension, and a well-established association between ID with/or without anaemia and poor outcome of PH patients, it is currently not clear, if ID and anaemia are merely a consequence of PH and relate to PH disease severity, or if they are implicated in the emergence and progression of PH as causative factors. Notably, different forms of iron dyshomeostasis and anaemia arise in patients suffering from PH [27]. The latter include IDA, ACD and complex forms of anaemia such as a combination of IDA/ACD. These forms of anaemia offer a diagnostic and therapeutic challenge for the clinician, as a precise classification of anaemia may be complicated and straightforward treatment suggestions are not available for complex forms of ID and/or anaemia thus far.

## Diagnosis and treatment of iron deficiency and anaemia in patients with pulmonary hypertension

Based on the accumulating evidence that ID and/or anaemia are frequently associated with PH, and that they affect mortality and morbidity of both, pre- and post-capillary PH patients, the impact of ID and/or anaemia correction on the course of PH was investigated [4, 5, 73]. However, most evidence originates from iron supplementation trials in patients with congestive heart failure (CHF), who frequently display post-capillary PH. Intravenous iron supplementation in CHF patients with ID improved functional capacity, quality of life and daily symptom burden [4]. Interestingly, anaemia was not a prerequisite for positive treatment response in that study, as both anaemic and non-anaemic CHF patients had a similar amelioration of symptoms following iron replacement. This suggests that ID in the absence of anaemia contributes to reduced exercise capacity in CHF. Iron supplementation attenuated the normal pulmonary vasoconstrictive response to hypoxia and improved some functional measures in PH patients regardless of the presence or absence of anaemia [5, 53, 61, 73, 74]. Thus, iron effects are rather based on alterations of cellular metabolism, mitochondrial function, muscle homeostasis or immune signalling. In pre-capillary PH iron supplementation has been tested in small patient cohorts with IPAH [5, 73]. Therapeutic correction of ID resulted in improvement of exercise endurance time, aerobic capacity and quality of life [5, 73]. These observations raise the question if a liberal iron supplementation strategy in PH patients with ID is of general benefit. In this context, it has to be mentioned that both, unbiased oral and intravenous iron administration, harbour limitations and risks in these patients. First, the exact pathophysiology of PH and PAH, especially the contribution of iron dyshomeostasis, is only partly understood and the consequences of therapeutic modifications of iron homeostasis in these individuals are largely elusive. Secondly, the diagnosis of the specific mode of ID, either absolute or functional or a combination of both is limited by diagnostic uncertainties and lack of good biomarkers specifically in the setting of chronic inflammation [12, 75, 76].

To date, clinical testing for ID mainly relies on the measurement of TSAT and serum ferritin concentration, which results in an inaccurate definition of ID and classification of anaemia in both, the clinical practice and currently available studies [4, 27]. TSAT and serum ferritin are not only altered by iron availability, but also various other triggers, including inflammatory processes and infection. For instance, a ferritin level between 30–100 µg/L may be indicative of appropriate iron availability in patients without signs of inflammation, whereas in patients with ongoing inflammatory processes the same value may be associated with functional ID [12, 77]. In this context, the measurement of sTFR, the sTFR/log ferritin index (sTFRF) and serum hepcidin may foster a more precise characterization of specific types of ID. Still, cut-offs and used parameters to best define ID in PH patients in the clinical practice are still controversial, which is reflected by a highly heterogenous definition of ID in various clinical trials (Table 2). A detailed evaluation of this topic was recently published by our working group [27].

**Table 2:** Most commonly used definitions of iron deficiency in patients with pulmonary hypertension

ID definitions	Serum ferritin (µg/L)	TSAT (%)	sTFR (mg/L)	sTFRF index
ID 1	< 30	< 16	–	–
ID 2	< 100	< 20	–	–
ID 3	<100		–	–
	or 100–299	If < 20		
ID 4	–	–	> 4.5 (female) > 5.0 (male)	–
ID 5	–	–	–	> 3.2 if CRP < 0.5 mg/dL > 2.0 if CRP > 0.5 mg/dL

*ID* iron deficiency, *TSAT* transferrin saturation, *CRP* C-reactive protein, *sTFR* soluble transferrin receptor, *sTFRF index* soluble transferrin receptor/log serum ferritin index; for a detailed evaluation of this topic refer to [27]

The issue of differentiating absolute from functional ID is from great clinical relevance because different therapy strategies would be applicable [1]. While iron supplementation is likely to be beneficial in situations of absolute ID, adding surplus iron to patients with inflammation-driven functional iron retention may be ineffective or even detrimental in certain conditions. Adverse effects of iron supplementation may include an impairment of the immune-surveillance or feeding of circulating pathogens leading to aggravation of infectious diseases, a hypothetic but not well-studied effect on malignant cell proliferation or the emergence of toxic iron effects via increased ROS/RNS production leading to cellular damage [11]. Consequently, available data on iron supplementation in PH patients have to be interpreted with caution and current evidence urges further investigation prior to the implementation of liberal iron supplementation strategies in PH patients. Third, and most importantly, we need to specifically identify those patients who will benefit from iron supplementation along with the definition of therapeutic start and endpoints.

Similarly to ID, anaemia has a high prevalence and is associated with increased disease severity and poor clinical outcome in PH patients [27, 28, 32]. The pathobiology of anaemia in PH is complex as various triggers including hypoxia, chronic blood loss, renal dysfunction, malnutrition, ID, chronic inflammation or recurrent infections are frequently found in PH. Consequently, PH patients represent various forms of anaemia, including ACD, IDA, IDA + ACD and non-classifiable/multifactorial forms of anaemia and the categorization and treatment of anaemia is complicated in these individuals [27]. To address this issue, additional parameters have been suggested to evaluate iron homeostasis in patients with PH. First, serum hepcidin measurements may help to predict response to oral iron treatment [13, 78]. Secondly, serum quantification of soluble transferrin receptor (sTFR) reflects cellular iron demand in the bone marrow [5, 27, 79]. The combination of sTFR and serum ferritin measurement permits the calculation of the sTFR/logferritin (sTFRF) index, which is currently considered as a clinical robust parameter to characterize iron homeostasis and anaemia, as it takes cellular iron demand and iron storage into account and may be corrected according to systemic signs of inflammation [12, 27]. Additionally, red cell indices as well as red cell distribution width (RDW) and zinc-protoporphyrin (Zn-pp) levels may help to identify functional ID at an earlier stage and may foster a diagnosis of functional ID prior to the emergence of anaemia or support the diagnosis of ACD [77, 78, 80]. Table 3 proposes a potential concept for clinicians to specify anaemia in PH patients.

**Table 3:** Classification of anaemia in pulmonary hypertension (adapted from [3])

	IDA	ACD	IDA + ACD	Unclassified anaemia
Haemoglobin	< 120 g/L for woman < 130 g/L for men			
Serum ferritin	< 30 µg/L	> 100 µg/L or 30–100 µg/L with reduced sTFRF index	30–100 µg/L	> 30 µg/L
TSAT	< 20%	< 20%	< 20%	Variable
sTFRF index	> 2	< 1 if serum ferritin 30–100 µg/L	> 2	1–2
Serum hepcidin	Reduced to below detection limit	Increased above normal	Normal or decreased	Variable

*IDA* iron deficiency anaemia, *ACD* anaemia of chronic disease, *IDA + ACD* a combination of IDA and ACD, *sTFRF index* soluble transferrin receptor log ferritin index, *TSAT* transferrin saturation

## Conclusion and perspective

Accumulating evidence suggests a pivotal role of iron homeostasis and anaemia in the pathophysiology, progression and prognosis of PH. Across all entities of PH, disturbances of iron homeostasis and anaemia are frequently observed, thus may represent a relevant contributor to disease severity and act as a potential treatment target in these patients. Still, due to the lack of clinically useful biomarkers, which precisely distinguish functional and true ID, and the lack of proper powered prospective randomized interventional trials in all different PH categories, iron supplementation has to be indicated with caution in PH patients. Systemic or local inflammation alters iron handling and puts patients at risk to suffer from potential detrimental effects of iron treatment. On the contrary, iron substitution may be safe and efficient in patients with true ID or IDA and it is important to screen for those conditions, as their treatment significantly improves PH patients’ morbidity and mortality. Consequently, the understanding of the interconnection of iron homeostasis, anaemia and pulmonary hypertension is a matter of high relevance, and ongoing scientific progress in the field offers the opportunity to close current knowledge gaps and provide new treatment options for PH patients.

## Methodology

We collected data and references with PubMed, MEDLINE, Google, and Google Scholar. The following keywords were used for literature search: anaemia, iron homeostasis, iron deficiency anaemia, anaemia of chronic disease, pulmonary hypertension, hypoxia.

## Abbreviations

ACD: Anaemia of chronic disease
AI: Anaemia of inflammation
BMP: Bone morphogenetic protein
BMPR2: Bone morphogenetic protein type-II receptor
CHF: Congestive heart failure
CTEPH: Chronic thromboembolic pulmonary hypertension
DMT1: Divalent metal transporter 1
DPG: Diastolic pressure gradient
EPO: EPO
ERFE: Erythroferrone
ESC: European Society of Cardiology
ERS: European Respiratory Society
Fe: Iron
FeTf: Iron loaded transferrin
FPN1: Ferroportin 1
FTN: Ferritin
GDF15: Growth differentiation factor 15
KC: Keratinocyte chemoattractant
LAST1: HIPPO central component large tumour suppressor 1
HAMP: Hepcidin antimicrobial peptide
HCP 1: Heme carrier protein 1
HFE: Human hemochromatosis gene
HGF: Hepatocyte growth factor
HIF: Hypoxia-inducible factor
HMOX1: Heme-oxygenase 1
HPAH: Heritable pulmonary arterial hypertension
HRG1: Haem transporter 1
ICAM1: Intercellular adhesion molecule 1
IDA: Iron-deficiency anaemia
IL1: Interleukin 1
IL6: Interleukin 6
IL10: Interleukin 10
IPAH: Idiopathic pulmonary arterial hypertension
dPAP: Diastolic pulmonary arterial pressure
mPAP: Mean pulmonary arterial pressure
MIF: Macrophage migration inhibitory factor
MPS: Mononuclear phagocyte system
MyD88: Molecular adaptor myeloid differentiation primary response protein 88
NF-kappaB: Nuclear factor kappa-light-chain-enhancer of activated B-cells
NTBI: Non-transferrin bound iron
PAH: Pulmonary arterial hypertension
PASMCs: Pulmonary artery smooth muscle cells
PAWP: Pulmonary arterial wedge pressure
PH: Pulmonary hypertension
PDGF-BB: Platelet-derived growth factor-BB
PVR: Pulmonary vascular resistance
RNS: Reactive nitrogen species
ROS: Reactive oxygen species
SMAD: Small mothers against decapentaplegic
SOD2: Mitochondrial superoxide dismutase 2
STAT3: Signal transducer and activator of transcription 3
sTFR: Soluble transferrin receptor
sTFRF: Soluble transferrin receptor log ferritin index
TGFbeta: Transforming growth factor-beta
TNFalpha: Tumour necrosis factor-alpha
TSAT: Transferrin saturation
VCAM1: Vascular cell adhesion molecule 1
VEGF: Vascular endothelial growth factor
WHO: World Health Organization
WSPH: World Symposium on Pulmonary Hypertension
ZIP14: Solute carrier family 39 zinc transporter
